# Human More Complex than Mouse at Cellular Level

**DOI:** 10.1371/journal.pone.0041753

**Published:** 2012-07-24

**Authors:** Alexander E. Vinogradov

**Affiliations:** Institute of Cytology, Russian Academy of Sciences, St.Petersburg, Russia; University of Saarland Medical School, Germany

## Abstract

The family of transcription factors with the C2H2 zinc finger domain is expanding in the evolution of vertebrates, reaching its highest numbers in the mammals. The question arises: whether an increased amount of these transcription factors is related to embryogenesis, nervous system, pathology or more of them are expressed in individual cells? Among mammals, the primates have a more complex anatomical structure than the rodents (e.g., brain). In this work, I show that a greater number of C2H2-ZFgenes are expressed in the human cells than in the mouse cells. The effect is especially pronounced for C2H2-ZF genes accompanied with the KRAB domain. The relative difference between the numbers of C2H2-ZF(-KRAB) genes in the human and mouse cellular transcriptomes even exceeds their difference in the genomes (i.e. a greater subset of existing in the genome genes is expressed in the human cellular transcriptomes compared to the mouse transcriptomes). The evolutionary turnover of C2H2-ZF(-KRAB) genes acts in the direction of the revealed phenomenon, i.e. gene duplication and loss enhances the difference in the relative number of C2H2-ZF(-KRAB) genes between human and mouse cellular transcriptomes. A higher amount of these genes is expressed in the brain and embryonic cells (compared with other tissues), whereas a lower amount - in the cancer cells. It is specifically the C2H2-ZF transcription factors whose repertoire is poorer in the cancer and richer in the brain (other transcription factors taken together do not show this trend). These facts suggest that increase of anatomical complexity is accompanied by a more complex intracellular regulation involving these transcription factors. Malignization is associated with simplification of this regulation. These results agree with the known fact that human cells are more resistant to oncogenic transformation than mouse cells. The list of C2H2-ZF genes whose suppression might be involved in malignization is provided.

## Introduction

The increase of biological complexity in the evolution is probably one of the most intriguing scientific problems. While complexity can easily be detected at the anatomical level (e.g., in the relative size and diversification of the nervous system), it is more difficult to analyze this phenomenon at the molecular and cellular levels [1], [2]. The amount of transcription factors (TFs) is a good candidate as possible indicator of cellular complexity because of regulatory role of TFs in the cell nucleus (similarly to the nervous system in the organism). The largest family of TFs in the mammalian genomes are genes with the C2H2 zinc finger domain (C2H2-ZF), many of them having also the Kruppel-associated box (KRAB) involved in chromatin remodelling [3].

This family is expanding in the evolution of vertebrates, reaching its highest numbers in the mammals [4], [5]. The question arises: whether an increased number of C2H2-ZF TFs is related to a greater complexity of embryogenesis, nervous system architecture, pathology (increased resistance to pathogens and stressful conditions) or a greater number of C2H2-ZF TFs are expressed in the individual cells? In the first case, the more complex organism is build from the same bricks (cells) as simpler organism and the increase of complexity rests entirely on the anatomical level. In the second case, the increase of anatomical complexity is associated with more complex intracellular regulation (i.e. more complex cells).

Among the mammals, the primates have a more complex anatomical structure than the rodents (e.g., brain [1]). To test the above hypotheses, I compare the numbers of expressed C2H2-ZF(-KRAB) genes in the transcriptomes of human and mouse tissues (in relation to the number of all expressed genes). In other words, the aim of this work is to check whether a greater number of various C2H2-ZF(-KRAB) genes is expressed on each expressed gene in the human cellular transcriptomes compared to the mouse transcriptomes. Besides the above-said significance of this problem for evolutionary biology, as the mouse is a paramount model for biomedical research, it is important to understand its differences from human at the cellular and molecular levels.

## Materials and Methods

The EST (expressed sequence tags) libraries were used because the EST technique was designed just for the qualitative determination of transcriptome repertoire [6]. Transcription factors are usually expressed in relatively low quantities [7], therefore the technique should be sensitive but accurate. The microarray technique uses an arbitrary expression threshold, which makes a problem for detection of low-expressed genes. The long sequence segments (several hundreds nucleotides) in the EST libraries exclude the problem of probe cross-hybridization (i.e. gene misidentification), which may arise for recent duplicates (with relatively low sequence divergence) in the case of microarray studies. (Many C2H2-ZF genes are recent duplicates.) Furthermore, in contrast to the microarray technique, the EST approach does not require the predefinition of genes and thus analyzes the transcriptome repertoire on the ‘as is’ basis. The efforts are taken by the libraries’ authors to reveal the maximum number of unique genes in the transcriptome at the expense of the accuracy of determination of their expression levels [6], [8]. (The latter parameter is not relevant for this study and was not used here.) There are many EST libraries from the various human and mouse tissues deposited in public domain, and the UniGene database regularly updates the mapping of these ESTs on the latest versions of the genomes [9].

The UniGene libraries containing more than 10,000 ESTs were used in this study. There were 170 human and 107 mouse libraries. There were no cancer libraries for mice, therefore the cancer factor was tested only for humans (where 69 cancer libraries were found). The InterPro database [10] was used for mapping of the C2H2-ZF and the KRAB domains to the human and mouse genes. In a special variant of analysis, the less complete but more homogenous (because of the automatic domain determination with the same algorithm) PFAM database [11] was used. The match between gene identifiers from different databases was made using the BioMart server (http://www.ensembl.org/biomart).

The human-mouse pairs of orthologous genes were determined as in [12]. Briefly, the human and mouse protein sequences were taken from the RefSeq database [9]. The matching of all human against all mouse (and vice versa) proteins was made using the Smith-Waterman algorithm implemented in the ‘ssearch’ program of the Fasta package with default Blosum50 matrix and the ‘shuffled’ calculation of statistical significance [13]. The reciprocal best hits of the longest proteins of the human-mouse gene pairs were treated as orthologous pairs. Only the unambiguous (1∶1) best hits were used.

The multivariate analysis of variance (ANOVA) was used for simultaneous estimation of the effect of the following factors: organism (human vs. mouse), embryogenesis (vs. non-embryogenesis), brain (vs. non-brain), cancer (vs. non-cancer), and tissue heterogeneity in an EST library (mixed tissues vs. non-mixed). To normalize the data for library size, the number of genes under question was divided by the total number of genes in a library. The type III sums of squares, which does not depend on the order, in which the tested parameters are introduced into the model, was used for the analysis. Type III quantifies the specific contribution of each parameter given that all other parameters have been accounted for. In other words, that component of the effect of a given parameter, which is independent of the effects of other parameters, is determined (i.e. which remains after the effects of other parameters were removed). The estimation of statistical significance of the effects was made using the Fisher F-value (the ratio of variance explained by a studied factor to error variance).

In another variant of analysis, the general linear model (GLM, a generalization of ANOVA, which allows simultaneous analyzing the effects of continuous and discrete variables) was used, with the total number of genes in an EST library being added as continuous variable into the model (instead of division of the number of genes under question by the total number of genes in a library, as in the main variant of analysis). Again, the type III sums of squares was used. In still other variant of analysis, the linear regression of the number of tested genes on the total number of genes in an EST library was used (instead of division of the number of genes under question by the total number of genes in a library), and the residuals of this regression were analyzed with ANOVA (as in the main variant of analysis). The analyses were done using the StatGraphics Centurion software package (Statpoint Technologies, Inc.).

## Results

### Main Phenomena

It is clearly seen that all studied factors (organism, embryogenesis, brain, and cancer), except for the tissue heterogeneity, have an effect (Table 1). The organismal factor (human vs. mouse) has the strongest effect, whereas the effect of the cancer factor was next to the strongest. The effects of the organismal factor and the cancer factor were stronger for the C2H2-ZF-KRAB genes than for the all C2H2-ZF genes (Table 1). The results obtained with the general linear model (GLM, with the total number of genes in an EST library being added into the model as continuous variable), and with the ANOVA analysis of the residuals of the linear regression of the number of studied genes on the total number of genes in an EST library were qualitatively the same (Tables S1 and S2). Also, the results obtained with determination of C2H2-ZF and KRAB domains using the more homogenous (but less complete) PFAM database were similar (Table S3).

**Table 1:** The results of multifactor analysis of variance (ANOVA) for the percentage of expressed C2H2-ZF(-KRAB) genes (in relation to all expressed genes).

Factor	all C2H2-ZF genes			C2H2-ZF-KRAB genes		
	*F*-ratio	*P*	Percentage of genes	*F*-ratio	*P*	Percentage of genes
human versus	48.11	10^−9^	3.20 (±0.26)	87.26	10^−16^	1.14 (±0.15)
mouse			2.44 (±0.31)			0.55 (±0.18)
embryo versus	11.08	0.001	3.03 (±0.26)	7.38	0.007	0.95 (±0.19)
non-embryo			2.60 (±0.31)			0.74 (±0.13)
brain versus	6.55	0.011	2.98 (±0.34)	8.05	0.005	0.94 (±0.19)
non-brain			2.66 (±0.31)			0.75 (±0.13)
cancer versus	20.63	10^−5^	2.53 (±0.35)	28.63	10^−6^	0.65 (±0.20)
non-cancer			3.11 (±0.23)			1.04 (±0.13)
mixed versus	0.54	0.46	2.89 (±0.43)	0.08	0.77	0.83 (±0.24)
non-mixed			2.75 (±0.18)			0.86 (±0.10)

F-ratios, significance levels, and the least squares mean percentages of these genes, with 95% confidence intervals.

The human cellular transcriptomes have about a 30% higher number of C2H2-ZF genes and about a 100% higher number of C2H2-ZF-KRAB genes (normalized to the total number of expressed genes, and with other tested factors being controlled) compared to the mouse transcriptomes (Table 1). The transcriptomes of non-cancer cells have about a 20% higher number of the C2H2-ZF genes and about a 60% higher number of the C2H2-ZF-KRAB genes than the cancer transcriptomes (Table 1).

### Comparison of the Difference in the Transcriptomes with the Difference in the Genomes

In the recent version of the human genome (according to the latest version of the InterPro database), there are 769 C2H2-ZF genes (among them 349 C2H2-ZF-KRAB genes). In the mouse genome, there are 616 C2H2-ZF genes (among them 252 C2H2-ZF-KRAB genes). In other words, in the human genome there are 25% more C2H2-ZF genes and 38% more C2H2-ZF-KRAB genes compared to the mouse genome. Thus, the human-mouse difference of the numbers of C2H2-ZF(-KRAB) genes in the cellular transcriptomes is even higher than their difference in the genomes (because a greater subset of the existing in the genome genes is expressed in the human cells compared to the mouse cells). The fact that there is a higher human-mouse difference of the numbers of C2H2-ZF(-KRAB) genes in the cellular transcriptomes than in the genomes excludes the explanation that this is a passive effect caused by transcription noise (assuming that a level of transcription noise in the human cells is not greater than in the mouse cells). This fact excludes also a possible problem of unequal coverage of human and mouse genomes in regard to discovery of C2H2-ZF(-KRAB) genes.

### Non-orthologous C2H2-ZF(-KRAB) Genes

In a special analysis, the non-orthologous C2H2-ZF(-KRAB) genes (i.e. genes originated by duplication or lost in the primate or rodent lineage after the separation of these lineages) were analyzed separately (Table 2). In regard to the organismal factor and the cancer factor, the effect was stronger for the non-orthologous C2H2-ZF(-KRAB) genes than for the all C2H2-ZF(-KRAB) genes (Tables 1 and 2). The difference between the human and mouse cellular transcriptomes for the non-orthologous C2H2-ZF(-KRAB) genes was even greater than for the all C2H2-ZF(-KRAB) genes. There are about 70% more non-orthologous C2H2-ZF genes and about 50% more non-orthologous C2H2-ZF-KRAB genes in the human genome compared with the mouse genome, whereas their difference in the cellular transcriptomes is about three-fold (Table 2).

**Table 2:** The results of multifactor analysis of variance (ANOVA) for the percentage of expressed non-orthologous C2H2-ZF(-KRAB) genes (in relation to all expressed genes).

Factor	all non-orthologous C2H2-ZF genes			non-orthologous C2H2-ZF-KRAB genes		
	*F*-ratio	*P*	Percentage of genes	*F*-ratio	*P*	Percentage of genes
human versus	138.96	10^−16^	0.90 (±0.12)	101.61	10^−16^	0.71 (±0.11)
mouse			0.29 (±0.14)			0.25 (±0.12)
embryo versus	6.22	0.013	0.67 (±0.16)	6.45	0.012	0.55 (±0.14)
non-embryo			0.52 (±0.11)			0.42 (±0.10)
brain versus	4.60	0.033	0.66 (±0.16)	5.19	0.024	0.54 (±0.14)
non-brain			0.53 (±0.11)			0.43 (±0.10)
cancer versus	33.83	10^−8^	0.42 (±0.16)	33.82	10^−7^	0.33 (±0.14)
non-cancer			0.77 (±0.11)			0.64 (±0.10)
mixed versus	0.56	0.45	0.56 (±0.20)	0.92	0.34	0.45 (±0.18)
non-mixed			0.63 (±0.08)			0.52 (±0.07)

F-ratios, significance levels, and the least squares mean percentages of these genes, with 95% confidence intervals.

Thus, the evolutionary turnover of C2H2-ZF(-KRAB) genes acts in the direction of the revealed phenomenon. In other words, gene duplication and loss in the primate vs. rodent lineages enhances the difference in the relative number of C2H2-ZF(-KRAB) genes between human and mouse cellular transcriptomes.

### Mixed and Separate Tissues

The heterogeneity of tissues in a library does not affect the ratio of the number of expressed C2H2-ZF(-KRAB) genes to the total number of expressed genes (Tables 1, 2, S1, S2 and S3). This fact suggests that the revealed effects are intracellular and are not stipulated by heterogeneity of cell types in the libraries. With other tested factors being controlled, the libraries of mixed tissues have on average a 25% higher total number of expressed genes compared with the homogenous libraries. Thus, in the heterogeneous libraries there is an increase both in the total number of expressed genes and in the number of expressed C2H2-ZF(-KRAB) genes in such a way that the percentage of C2H2-ZF(-KRAB) genes remains the same.

The effect was also checked in the separate tissues. For this purpose, the human-mouse libraries obtained from homologous tissues were selected (with at least three libraries from the non-cancer, non-embryo, non-mixed tissue for each human and mouse tissue). The higher ratio of the number of expressed C2H2-ZF(-KRAB) genes to the total number of expressed genes was observed for the each pair of homologous tissues (Tables S4 and S5). Although in some cases there was only marginal significance of this difference (probably because of a small number of libraries for each pair of tissues), in all cases the difference was consistently in the same direction (Tables S4 and S5). Totally for all pair-wise comparisons of these homologous tissues, the average human-mouse difference was highly significant (*P*<10^−3^).

The comparison of separate tissues within the same species seems so far premature because of a low number of libraries for each tissue (and this comparison is not important for the aim of this work). In the human, some tissues have a seemingly similar percentage of expressed C2H2-ZF(-KRAB) genes with the brain, whereas in the mouse this is not so (Tables S4 and S5). At least, one can conclude that when compared with all other tissues taken together, the brain shows a higher percentage of expressed C2H2-ZF(-KRAB) genes, with the organismal, embryogenesis and cancer factors being accounted for (Tables 1, S1, S2 and S3).

### Non C2H2-ZF Transcription Factors

In a separate analysis, the human genes mapped to “transcription factor activity” in the ‘Molecular Functions’ section of the Gene Ontology database [14] but not having the C2H2-ZF domain were determined in the EST libraries (there were 806 such genes in the human genome). Their percentage was slightly higher in the transcriptomes of cancer tissues compared to normal tissues (Table 3). In the brain, their percentage was lower compared with the non-brain tissues (Table 3). Thus, it is specifically the C2H2-ZF transcription factors whose repertoire is poorer in the cancer cells and richer in the brain.

**Table 3:** The results of multifactor analysis of variance (ANOVA) for the percentage of expressed human genes mapped to “transcription factor activity” in the ‘Molecular Functions’ section of the Gene Ontology database but not having C2H2-ZF domain (in relation to all expressed genes).

Factor	*F*-ratio	*P*	Percentage of genes
embryo versus	3.75	0.054	3.70 (±0.22)
non-embryo			3.51 (±0.15)
brain versus	37.47	10^−8^	3.38 (±0.20)
non-brain			3.82 (±0.15)
cancer versus	8.87	0.003	3.70 (±0.19)
non-cancer			3.51 (±0.15)
mixed versus	3.98	0.048	3.73 (±0.27)
non-mixed			3.47 (±0.11)

F-ratios, significance levels, and the least squares mean percentages of these genes, with 95% confidence intervals.

### Olfactory-receptor Genes (as an Additional Control for Transcription Noise in the EST Libraries)

As an additional control for possible transcription noise in the EST libraries, the numbers of genes with the olfactory-receptor domain were analyzed. This is a large gene family similar in gene amount and evolutionary turnover (gene duplication and loss) with the C2H2-ZF family [5], [15]. The ectopic expression of these genes in the non-olfactory tissues was reported [16]. I found 373 such genes in the human genome and 1099 genes in the mouse genome. (It is known that mice have many more olfactory-receptor genes than humans because rodents are olfactory specialists, whereas primates are visual specialists [15].) In spite of a high number of these genes in the genomes, the expression of only a negligible amount of them was detected (the studied libraries do not include the olfactory epithelium) (Table 4). Notwithstanding a roughly similar order of magnitude of the olfactory-receptor genes and the C2H2-ZF genes in the genome, in the cellular transcriptomes the number of olfactory-receptor genes was about 300-folds lower. (The expression of a few olfactory-receptor genes can be explained by their involvement in cellular chemoreception.) Notably, it was recently shown that in mammals the mRNA level and the protein level are well correlated [17].

**Table 4:** The results of multifactor analysis of variance (ANOVA) for the percentage of expressed olfactory-receptor genes (in relation to all expressed genes).

Factor	*F*-ratio	*P*	Percentage of genes
human versus	2.30	0.13	0.0047 (±0.0065)
mouse			0.0088 (±0.0076)
embryo versus	1.39	0.24	0.0049 (±0.0085)
non-embryo			0.0087 (±0.0059)
brain versus	5.39	0.02	0.0032 (±0.0084)
non-brain			0.0103 (±0.0058)
cancer versus	3.88	0.05	0.0037 (±0.0086)
non-cancer			0.0099 (±0.0057)
mixed versus	0.10	0.75	0.0076 (±0.0106)
non-mixed			0.0060 (±0.0044)

F-ratios, significance levels, and the least squares mean percentages of these genes, with 95% confidence intervals.

## Discussion

With the anatomical-level factors (embryogenesis and brain) and cancer factor being accounted for, a greater number of C2H2-ZF(-KRAB) genes is expressed on each expressed gene in the human cells compared with the mouse cells. This fact suggests that the increase of anatomical complexity in the evolution is accompanied by more complex intracellular regulation involving these transcription factors. In other words, a more complex organism is build from more complex cell “bricks”. The evolutionary turnover of C2H2-ZF(-KRAB) genes acts in the direction of the revealed phenomenon, i.e. gene duplication and loss enhances the difference in the relative number of C2H2-ZF(-KRAB) genes between human and mouse cellular transcriptomes. Malignization is associated with simplification of this regulation. It is specifically the C2H2-ZF(-KRAB) TFs whose repertoire is poorer in the cancer cells (and richer in the brain).

The C2H2-ZF-KRAB genes act mostly as chromatin-modulating (long-term) transcription repressors [18]. The higher number of them in the human transcriptome might be related to the finding that human cells have a greater fraction of tissue-specific genes (i.e. genes suppressed in most tissues) than mouse cells [2]. Importantly, the human cells are more resistant to oncogenic transformation and generally have higher metabolic stability [19]–[21], which agrees well with the results obtained in this work (because a lower number of C2H2-ZF genes are expressed in the cancer cells than in normal cells).

A detailed architecture of intracellular regulatory networks involving human C2H2-ZF(-KRAB) TFs is an intriguing avenue of further research. In particular, the increase of regulatory complexity associated with these genes may stipulate the resistance of human cells to malignization. It is possible that protein products of certain of them can be used as tumor suppressors. (The perspective candidates are shown in Tables S6 and S7.) The C2H2-ZF(-KRAB) TFs might be perspective candidates for molecular therapy because of their participation in deep layers of regulatory networks determining long-term cell state.

## Supporting Information

**Table S1** **The results of the general linear model (GLM, with the total number of expressed genes being added as continuous variable) for the number of expressed C2H2-ZF(-KRAB) genes.**
(PDF)

**Table S2** **The results of multifactor analysis of variance (ANOVA) for the residuals of the linear regression of the number of expressed C2H2-ZF(-KRAB) genes on the total number of expressed genes.** The residuals are negative and positive values distributed around zero mean.
(PDF)

**Table S3** **The results of multifactor analysis of variance (ANOVA) for the percentage of expressed C2H2-ZF(-KRAB) genes (in relation to all expressed genes) determined using the PFAM database.**
(PDF)

**Table S4** **The comparison of the percentages of expressed C2H2-ZF genes (in relation to all expressed genes) in the homologous human and mouse tissues.**
(PDF)

**Table S5** **The comparison of the percentages of expressed C2H2-ZF-KRAB genes (in relation to all expressed genes) in the homologous human and mouse tissues.**
(PDF)

**Table S6 (List A)** **The human C2H2-ZF genes presented in the transcriptome of normal tissues and absent in the cancer tissues.** (Ranking by the total EST count of a given gene normalized by the library sizes.).
(PDF)

**Table S7 (List B)** **The human C2H2-ZF genes overrepresented in the transcriptome of normal tissues compared with the cancer tissues.** (Ranking by the ratio of the EST count in the normal tissues to the count in the cancer tissues. Genes with the ratio above three-fold are shown. The counts were normalized by the number and the sizes of the EST libraries.)
(PDF)
