# Supplementary material for: Human More Complex than Mouse at Cellular Level
Source: PLoS One. 2012 Jul 24;7(7):e41753. doi: 10.1371/journal.pone.0041753 (PMC3404003; doi:10.1371/journal.pone.0041753)
Supplement: Table S1 — The results of the general linear model (GLM, with the total number of expressed genes being added as continuous variable) for the number of expressed C2H2-ZF(-KRAB) genes. (PDF) [file pone.0041753.s001.pdf]

Table S1. The results of the general linear model (GLM, with the total number of expressed genes being added as continuous variable) for the number of expressed C2H2-ZF(-KRAB) genes.

Factor	all C2H2-ZF genes			C2H2-ZF-KRAB genes		
	F-ratio	*P*	Number of genes	*F*-ratio	*P*	Number of genes
human versus mouse	44.41	10^{-9}	158.6 (±11.9) 125.0 (±13.8)	76.91	10^{-16}	58.8 (±7.6) 30.4 (±8.9)
embryo versus non-embryo	11.65	0.001	151.8 (±15.3) 131.8 (±10.7)	5.65	0.02	49.4 (±9.8) 40.1 (±6.9)
brain versus non-brain	6.37	0.011	148.8 (±15.2) 134.8 (±10.6)	7.35	0.007	49.4 (±9.7) 39.8 (±6.8)
cancer versus non-cancer	12.10	0.001	131.7 (±15.5) 151.9 (±10.4)	19.70	10^{-5}	36.3 (±9.9) 52.9 (±6.7)
mixed versus non-mixed	0.03	0.85	142.6 (±19.3) 141.0 (±8.0)	0.45	0.5	42.7 (±12.3) 46.5 (±5.1)

F-ratios, significance levels, and the least squares mean numbers of these genes, with 95% confidence intervals.
