# Supplementary material for: Human More Complex than Mouse at Cellular Level
Source: PLoS One. 2012 Jul 24;7(7):e41753. doi: 10.1371/journal.pone.0041753 (PMC3404003; doi:10.1371/journal.pone.0041753)
Supplement: Table S2 — The results of multifactor analysis of variance (ANOVA) for the residuals of the linear regression of the number of expressed C2H2-ZF(-KRAB) genes on the total number of expressed genes. The residuals are negative and positive values distributed around zero mean. (PDF) [file pone.0041753.s002.pdf]

Table S2. The results of multifactor analysis of variance (ANOVA) for the residuals of the linear regression of the number of expressed C2H2-ZF(-KRAB) genes on the total number of expressed genes. The residuals are negative and positive values distributed around zero mean.

Factor	all C2H2-ZF genes			C2H2-ZF-KRAB genes		
	F-ratio	*P*	Residuals	*F*-ratio	*P*	Residuals
human versus mouse	41.41	10^{-8}	18.35 (±15.61)<br>-13.45 (±18.23)	72.58	10^{-16}	10.21 (±10.03)<br>-16.85 (±11.71)
embryo versus non-embryo	11.03	0.001	12.20 (±20.24)<br>-7.31 (±14.15)	5.15	0.02	0.96 (±13.01)<br>-7.60 (±9.09)
brain versus non-brain	6.67	0.010	9.60 (±20.04)<br>-4.71 (±13.98)	7.69	0.005	1.62 (±12.88)<br>-8.26 (±8.98)
cancer versus non-cancer	10.24	0.001	-6.71 (±20.45)<br>11.60 (±13.59)	16.99	10^{-4}	-10.89 (±13.16)<br>4.25 (±8.73)
mixed versus non-mixed	0.00	0.95	2.15 (±25.35)<br>2.74 (±10.48)	0.93	0.34	-6.03 (±16.29)<br>-0.61 (±6.73)

F-ratios, significance levels, and the least squares mean residual values, with 95% confidence intervals.
