# Supplementary material for: Human More Complex than Mouse at Cellular Level
Source: PLoS One. 2012 Jul 24;7(7):e41753. doi: 10.1371/journal.pone.0041753 (PMC3404003; doi:10.1371/journal.pone.0041753)
Supplement: Table S3 — The results of multifactor analysis of variance (ANOVA) for the percentage of expressed C2H2-ZF(-KRAB) genes (in relation to all expressed genes) determined using the PFAM database. (PDF) [file pone.0041753.s003.pdf]

Table S3. The results of multifactor analysis of variance (ANOVA) for the percentage of expressed C2H2-ZF(-KRAB) genes (in relation to all expressed genes) determined using the PFAM database.

Factor	all C2H2-ZF genes			C2H2-ZF-KRAB genes		
	F-ratio	P	Percentage of genes	*F*-ratio	*P*	Percentage of genes
human versus mouse	43.01	10^{-9}	2.90 (±0.33) 2.21 (±0.39)	86.65	10^{-16}	1.24 (±0.21) 0.63 (±0.24)
embryo versus non-embryo	11.46	0.001	2.77 (±0.43) 2.34 (±0.30)	7.10	0.008	1.04 (±0.27) 0.83 (±0.19)
brain versus non-brain	8.17	0.005	2.73 (±0.39) 2.39 (±0.30)	8.55	0.004	1.05 (±0.27) 0.83 (±0.18)
cancer versus non-cancer	12.14	10^{-4}	2.27 (±0.43) 2.84 (±0.29)	26.96	10^{-6}	0.74 (±0.27) 1.13 (±0.18)
mixed versus non-mixed	0.35	0.64	2.63 (±0.54) 2.48 (±0.22)	0.11	0.74	0.92 (±0.34) 0.95 (±0.14)

F-ratios, significance levels, and the least squares mean percentages of these genes, with 95% confidence intervals.
