# Supplementary material for: Human More Complex than Mouse at Cellular Level
Source: PLoS One. 2012 Jul 24;7(7):e41753. doi: 10.1371/journal.pone.0041753 (PMC3404003; doi:10.1371/journal.pone.0041753)
Supplement: Table S4 — The comparison of the percentages of expressed C2H2-ZF genes (in relation to all expressed genes) in the homologous human and mouse tissues. (PDF) [file pone.0041753.s004.pdf]

Table S4. The comparison of the percentages of expressed C2H2-ZF genes (in relation to all expressed genes) in the homologous human and mouse tissues.

Tissue	Percentage of genes		P for difference
	human	mouse	
brain	3.17 (±0.35)	2.59 (±0.31)	0.01
kidney	3.15 (±0.67)	1.82 (±1.24)	0.02
liver	2.66 (±2.81)	1.24 (±2.92)	0.05
pancreas	2.95 (±0.68)	1.73 (±3.36)	0.12
testis	3.57 (±1.02)	1.83 (±1.09)	0.01
