# Supplementary material for: Human More Complex than Mouse at Cellular Level
Source: PLoS One. 2012 Jul 24;7(7):e41753. doi: 10.1371/journal.pone.0041753 (PMC3404003; doi:10.1371/journal.pone.0041753)
Supplement: Table S5 — The comparison of the percentages of expressed C2H2-ZF-KRAB genes (in relation to all expressed genes) in the homologous human and mouse tissues. (PDF) [file pone.0041753.s005.pdf]

Table S5. The comparison of the percentages of expressed C2H2-ZF-KRAB genes (in relation to all expressed genes) in the homologous human and mouse tissues.

Tissue	Percentage of genes		P for difference
	human	mouse	
brain	1.34 (±0.23)	0.71 (±0.13)	$2*10^{-5}$
kidney	1.15 (±0.49)	0.57 (±1.04)	0.09
liver	0.86 (±1.75)	0.25 (±1.85)	0.09
pancreas	0.93 (±0.42)	0.47 (±3.22)	0.14
testis	1.62 (±0.68)	0.62 (±0.40)	0.01
