# Supplementary material for: Human More Complex than Mouse at Cellular Level
Source: PLoS One. 2012 Jul 24;7(7):e41753. doi: 10.1371/journal.pone.0041753 (PMC3404003; doi:10.1371/journal.pone.0041753)
Supplement: Table S6 (List A) — The human C2H2-ZF genes presented in the transcriptome of normal tissues and absent in the cancer tissues. (Ranking by the total EST count of a given gene normalized by the library sizes.). (PDF) [file pone.0041753.s006.pdf]

Table S6 (List A). The human C2H2-ZF genes presented in the transcriptome of normal tissues and absent in the cancer tissues. (Ranking by the total EST count of a given gene normalized by the library sizes.)

EntrezGene	Count
7768	37.58
346157	36.26
50943	34.87
646	30.62
65243	25.12
128209	20.59
285346	16.10
56979	13.85
389549	12.72
654254	9.92
201516	9.46
7638	9.09
728116	8.18
10661	8.15
347741	7.81
168417	7.01
7652	5.52
7634	4.97
148198	4.21
170960	4.18
100287429	4.17
57615	3.69
136259	3.53
219578	3.17
442319	2.85
90827	2.62
100132396	2.27
342933	2.26
728361	2.26
158506	1.70
221527	1.42
100131390	1.14
