# Supplementary material for: Human More Complex than Mouse at Cellular Level
Source: PLoS One. 2012 Jul 24;7(7):e41753. doi: 10.1371/journal.pone.0041753 (PMC3404003; doi:10.1371/journal.pone.0041753)
Supplement: Table S7 (List B) — The human C2H2-ZF genes overrepresented in the transcriptome of normal tissues compared with the cancer tissues. (Ranking by the ratio of the EST count in the normal tissues to the count in the cancer tissues. Genes with the ratio above three-fold are shown. The counts were normalized by the number and the sizes of the EST libraries.) (PDF) [file pone.0041753.s007.pdf]

Table S7 (List B). The human C2H2-ZF genes overrepresented in the transcriptome of normal tissues compared with the cancer tissues. (Ranking by the ratio of the EST count in the normal tissues to the count in the cancer tissues. Genes with the ratio above three-fold are shown. The counts were normalized by the number and the sizes of the EST libraries.)

EntrezGene	Ratio
55079	30.49
113835	22.96
7597	21.15
147929	16.58
84891	14.55
255877	13.87
342908	13.71
57573	11.26
64919	10.63
169841	10.22
79673	8.50
347344	8.14
643836	7.92
7789	7.55
339318	6.94
53336	6.86
7738	6.73
163115	6.67
148254	6.52
203523	6.42
11279	6.29
84436	6.24
284349	6.24
729288	6.22
84107	6.18
54796	6.06
158431	5.94
219749	5.91
92283	5.90
79891	5.65
284370	5.62
7745	5.40
6299	5.39
7718	5.33
151126	5.30
167465	5.20
9745	5.08
252884	4.99
7762	4.97
100289635	4.88
390980	4.77
7710	4.70
79898	4.69
284312	4.69

26137	4.68
25925	4.63
158399	4.56
7592	4.54
140883	4.50
7730	4.46
170691	4.38
125893	4.38
7490	4.22
7562	4.19
148266	4.13
80032	4.12
641339	4.12
388536	4.07
79973	4.07
7539	4.05
27107	4.05
282890	4.01
339324	4.01
126070	3.99
83744	3.95
9668	3.95
7700	3.94
11244	3.93
9839	3.88
80108	3.86
80095	3.86
6935	3.81
163255	3.79
64288	3.78
284391	3.76
124961	3.67
57711	3.60
64376	3.59
199704	3.59
168374	3.54
63978	3.52
8328	3.52
162993	3.49
57336	3.49
221895	3.48
7773	3.47
163051	3.43
163049	3.42
158586	3.38
65251	3.38
6297	3.28
51276	3.26
148268	3.25
285676	3.24
